# Food hygiene practices and its associated factors among model and non model households in Abobo district, southwestern Ethiopia: Comparative cross-sectional study

**DOI:** 10.1371/journal.pone.0194391

**Published:** 2018-04-05

**Authors:** Akoma Okugn, Demelash Woldeyohannes

**Affiliations:** 1 Department of Nursing, Gambella College of Health Science, Gambella, Ethiopia; 2 Department of Nursing, Collage of Medicine and Health Science, Madda Walabu University, Bale Goba, Ethiopia; Agricultural University of Athens, GREECE

## Abstract

**Background:**

In developing country most of human infectious diseases are caused by eating contaminated food. Estimated nine out ten of the diarrheal disease is attributable to the environment and associated with risk factors of poor food hygiene practice. Understanding the risk of eating unsafe food is the major concern to prevent and control food borne diseases. The main goal of this study was to assessing food hygiene practices and its associated factors among model and non model households at Abobo district.

**Methods:**

This study was conducted from 18 October 2013 to 13 June 2014. A community-based comparative cross-sectional study design was used. Pretested structured questionnaire was used to collect data. A total of 1247 households (417 model and 830 non model households) were included in the study from Abobo district. Bivariate and multivariate logistic regression analysis was used to identify factors associated with outcome variable.

**Results:**

The study revealed that good food hygiene practice was 51%, of which 79% were model and 36.70% were non model households. Type of household [AOR: 2.07, 95% CI: (1.32–3.39)], sex of household head [AOR: 1.63, 95% CI: (1.06–2.48)], Availability of liquid wastes disposal pit [AOR: 2.23, 95% CI: (1.39,3.63)], Knowledge of liquid waste to cause diseases [AOR: 1.95, 95% (1.23,3.08)], and availability of functional hand washing facility [AOR: 3.61, 95% CI: (1.86–7.02)] were the factors associated with food handling practices.

**Conclusion:**

This study revealed that good food handling practice is low among model and non model households. While type of household (model versus non model households), sex, knowledge of solid waste to cause diseases, availability of functional hand washing facility, and availability of liquid wastes disposal pit were the factors associated with outcome variable. Health extension workers should play a great role in educating households regarding food hygiene practices to improve their knowledge and practices of the food hygiene.

## Background

Eating contaminated food can cause infection in human [1]. Although most of the waterborne and food borne diseases, illnesses and deaths are never reported. The highest epidemics of cholera of the 2010–2011 outbreaks in Haiti alone caused more than 500,000 cases of illness and 7,000 deaths [2, 3].

Food is dangerous and causes diseases if not processed, prepared and maintained in sanitary and safe conditions. Diseases such as diarrhea, Typhoid fever, cholera, Amoebiasis, Tapeworm, Anthrax, and Bovine are transmitted to man through contaminated unsafe food. When foodstuff has been in contact with hazardous toxic chemicals during food production, processing, storage and handling it can also lead to chemical food poisoning. Foodstuffs contaminated by microbial pathogens or toxic chemicals as a result of poor handling are dangerous to human beings [4].

A lot of world’s poorest people die from preventable diseases each year which resulted from lack of good food hygiene [5].The problem of food borne disease is more serious among the rural communities because of their low level of awareness and generally the prevailing poor and unhygienic environment [4].

In Gambella region 70.9% water supply was from the unimproved source. The region obtained only 29.1% water supply from improved types of technologies. Improved source of water is the main determinant of food hygiene practices [6]. The same to water source, household latrine coverage is also another determinant for food hygiene practices. In this region, the coverage of household latrine was only25.7% [7]. As result, food borne diseases are found at the top causes of morbidity in the region [8]. The national highest diarrhea prevalence among children is 23% which is found in Gambella regions [9].

Morbidity and mortality from diarrhea diseases in our country and in Gambella region are still high which are related to food hygiene practices [10]. In Gambella region, most of the households are still not practicing good hygienic practice during food handling [11].

In Ethiopia, particularly in Gambella region, there is no study conducted on practices food hygiene practices and its associated factors in both model and non model household. So, this study was conducted to identify those gaps of food hygiene practices and its associated factors at model and non model households. The finding of this research will be used by regional health bureau, researchers, and other program implementers.

## Methodology

### Study design and study period

The community-based comparative cross-sectional study design was conducted from October 18, 2013, to June 13, 2014 at Abobo district.

### Study area

Abobo district is found in Gambella People’s Regional state and822 Kms far from Addis Ababa and 45 Kms far from Gambella town, the capital of Gambella Regional state. It has rural Kebeles (lowest administrative unit) administration and urban Kebeles [12]. The district has a total of 1257 households and 11,951 total populations. Among those households 417 households were the model and 835 households were non model [13].

### Population

#### Source population and study population

All model and non model households in the Abobo district were considered as the source population and study population of the study.

### Sample size determination

The sample size was not calculated because all model and non model households in the district were included in the study. Total number of the study participants was 1252, from total number of the study participants 417 were model households and 835 were non model households.

### Sampling procedures

All model and non model households in the district were included in the study. Those model and non model households were taken from registration book of Health Extension workers (community workers at the district).

### Data collection procedures

#### Data collection instruments

Structured questionnaire was used to collect data. The questioner was prepared after reviewing relevant literatures. The questioner was first prepared in English and translated to local language (Anywa), then translated back to English to check its consistence.

## Data quality control

The data were checked for completeness and consistency. Data collection process and other field works were closely supervised by supervisors and investigators. Double data entry was done and the questionnaire was pre-tested in Gambella district before starting the actual data collection. After pretest of the questionnaire minor corrections were made on the questions including editing the questions to make easily understandable by data collectors and supervisors. For data collectors and supervisors three days training were given on the objective of the study and on how they will approach the study participants.

### Ethical considerations

Ethical clearance was approved from the ethical committee of College of Medicine and Health Sciences of Bahir Dar University and then formal letters from Bahir Dar University was given to Gambella Regional Health Bureau for their collaboration. And Gambella Regional Health Bureau wrote a letter to Abobo district health office. Written consent was obtained from each study participants after explaining the purpose of the study. Confidentiality of the study participants was secured by not recording the identification of study participants, questionnaires were kept locked, and data collectors kept the information strictly confidential. And only voluntary study participants were included in the study.

### Data analysis

The data entry was done by using EPI Info 3.5 and analyzed by SPSS Version 20. To summarize data frequencies and cross tabulations were used. Data presentation was done by using tables. Bivariate and multivariate logistic regression analyses were used to identify the factors associated with food hygiene practices. The variables with the p-value less than or equal to 0.2 in the binary logistic regression analysis were considered for multivariate logistic regression analysis. The variables with probability values (p) ≤ 0.05 were considered statistically significant with outcome variables. Stepwise method was used. Finally, the variables which had significant association with outcome variables were identified on the basis of adjusted odd ratio with 95% CI and p-value.

### Operational definition

#### Model household

Households, who attended above 75% training hours of the sixteen health extension packages, correctly implement at least the sixteen health extension packages, correctly answer three or more out of five questions of training in the presence of health extension supervisor and then certified as model households.

#### Good food hygiene practice

If a household practiced > = three proper food hygiene practices from total of six question were considered as good food hygiene practices

#### Not good food hygiene practice

If a household practiced < three proper food hygiene practices from out of six practice questions were considered as not good food hygiene practices.

### Inclusion and exclusion criteria

#### Inclusion criteria

Mothers or household heads (both model and non model households) that lived for more than 6 months in the study area and voluntary to participate in the study was included in the study.

#### Exclusion criteria

Mothers or household heads (model or non model) that were sick and unable to respond the interview were excluded from the study. However, there were no such households obtained during the interview.

## Results

### Characteristics of study participants

Out of the total 1247 households, one third of study participants were model households 417(33.40%) whereas two third of study participants were non model households and females 830(66.6%) with the response rate of 99.60%. The mean age of study participants was 34(±8.30) years. Among study participants the majorities of them were married 670(80.30%), Protestants religion followers 897(71.90%) and Anywa ethnicity 714(57.30%). Less than half of the study participants were housewives and illiterate, 558 (44.70%) and 570(45.70%) respectively. More than three fourth of the study participants had greater than five family members 1052(84.36%) **(Table 1)**.

**Table 1:** Socio demographic characteristics of respondents, Abobo district, Gambella People National Regional State, April 2014(n = 1247).

	Type of household		
Variables	Model HH(n = 417)	Nonmodel HH(n = 830)	Total (n = 1247)
	Number (%)	Number (%)	Number (%)
**Sex**			
Female	336(80.60)	493(59.40)	829(66.50)
Male	81(19.40)	337(40.60)	418(33.50)
**Age**			
18–27	124(29.70)	98(11.80)	222(17.80)
28–37	184(44.10)	443(53.40)	627(50.30)
38–47	77(18.50)	211(25.40)	288(23.10)
> = 48	32(7.70)	78(9.40)	110(8.80)
**Mean (SD)**			34 ±8.30
**Education Level**			
Illiterate	182(43.60)	388(46.70)	570(45.70)
Only read and write	16(3.80)	30(3.60)	46(3.70)
Primary school(1–8)	188(45.10)	344(41.40)	532(42.70)
Secondary school(9–12) and above	31(7.40)	68(8.20)	99(7.90)
**Religion**			
Orthodox	131(31.40)	61(7.30)	192(15.40)
Protestant	198(47.50)	699(84.20)	897(71.90)
Catholic	13(3.10)	20(2.40)	33(2.60)
Muslim	75(18.00)	50(6.00)	125(10.00)
**Marital status**			
Single	9(2.20)	11(1.30)	20(1.60)
Married	331(79.40)	670(80.70)	1001(80.30)
Divorced	15(3.60)	26(3.10)	41(3.30)
Widowed	22(5.30)	51(6.10)	73(5.90)
Separated	40(9.60)	72(8.70)	112(9.00)
**Occupation**			
Farmer	119(28.50)	408(49.20)	527(42.30)
Merchant	38(9.10)	86(10.40)	124(9.90)
Housewife	319(38.40)	239(57.30)	558(44.70)
Other*	17(2.00)	21(5.00)	38(3.00)
**Ethnicity**			
Anywa	58(13.90)	656(79.00)	714(57.30)
Kembata	139(33.30)	67(8.10)	206(16.50)
Amhara	114(27.30)	46(5.50)	160(12.80)
Other**	106(25.40)	61(7.30)	167(13.40)
**Family size in** **Number**			
<5	329(78.89)	723(87.12)	1052(84.36)
> = 5	88 (21.11)	107(1.88)	195 (15.64)
**HHs income per** **month in Birr**			
< = 600	318(76.2)	781(94.1)	1099(88.20)
>600	99(23.70)	49(5.90)	148(11.80)

* Government employee, Daily Workers

**Oromo, Hadiya, Wolayita

### Food hygiene practices

Among the households which included in the study 665(51.00%) of them had good food hygiene practice; of which 330(79.00%) were model and 350 (36.70%) were non model households. From total household 56 (4.50%) of which 54 (12.90%) model and 2 (0.20%) were non model households prepared their food in the separated kitchen and the rest prepared their foods outdoor and other places **(Table 2)**.

**Table 2:** Food hygiene practices of the respondents, Abobo District Gambella People National Regional State, April 2014(n = 1247).

Variables	Type of house hold		Total (n = 1247)
	Model HH (n = 417)	Non-model HH (n = 830)	
	Number (%)	Number (%)	Number (%)
**Place where food is prepared**			
In the separated kitchen	54(12.90)	2(0.20)	56(4.50)
Not in separated kitchen*	363(87.10)	828(99.8)	1191(95.50)
**Place for keeping catering utensils after cooking**			
Kept on the shelf	298(71.50)	276(33.30)	574(46.00)
Not on the shelf**	119(28.50)	554(66.70)	673(54.00)
**Type of water used to prepare food**			
Improved	368(88.20)	536(64.60)	904(72.50)
Unimproved	49(11.80)	294(35.40)	343(27.50)
**Keeping cooked and raw foods separately**			
Yes	373(89.40)	663(79.90)	1036(83.10)
No	44(10.60)	167(20.10)	211(16.90)
**Covering of all cooked foods**			
Yes	373(89.40)	663(79.90)	1036(83.10)
No	44(10.60)	167(20.10)	211(16.90)
**Washing hand always before preparing foods**			
Yes	324(77.70)	333(40.10)	657(52.70)
No	93(22.30)	497(59.90	590(47.30)
**Over all food hygiene handling practices**			
Good	330(79.00)	305(36.70)	635(51.00)
Not good	87(21.00)	525(63.30)	612(49.00)

* = no chimney, not clean, shared and outdoor

** = kept on ground and kept on local barn

### Factors associated with food hygiene practices

In bivariate logistic regression analysis the variables; type of households, sex of household head, family size, knowledge on the cause of diarrheal diseases, using leftover food, availability of functional hand washing facility and availability of solid wastes disposal pit had significant association with food hygiene practices. However, in multivariate logistic regression analysis household type (model versus non model households), sex, knowledge of liquid waste to cause diseases, availability of functional hand washing facility, availability of latrine and availability of liquid wastes disposal pit were significantly association with food hygiene practices.

Model households were 2.07 times more likely to practice good food hygiene practices than their counterparts [AOR: 2.07, 95% CI: (1.32–3.39)]. Female household heads were 1.63 times more likely practices good food hygiene practices than male household heads [AOR: 1.63, 95% CI: (1.06–2.48)]. Moreover, households who had functional hand washing facility were 2.23 times more likely to practices good food hygiene practices than the households who had not functional hand washing facility [AOR: 3.61,95%CI: (1.86–7.02)]**(Table 3)**.

**Table 3:** Factors associated with food hygiene practices Abobo District, Gambella People National Regional State, April 2014(n = 1247).

Characteristics	Status of food hygiene practices			
	Both households (n = 1247)			
	Good	Not good	COR	AOR
**Type of household**				
Model	330(79.10)	87(20.90)	6.53(4.91,8.68)	2.09(1.30,3.37) ^a^
None model	305(36.70)	525(63.30)	1.00	1.00
**Sex**				
Female	508(61.30)	321(38.70)	3.63(2.80,4.70)	1.63(1.06,2.48) ^b^
Male	127(30.40)	291(69.60)	1.00	1.00
**Age**				
18–27	174(78.40)	48(21.60)	4.43(0.01,5.54)	
> = 28	461(45.00)	564(55.00)	1.00	
**Family size**				
<4	287(43.50)	373(56.50)	0.53(0.42,0.67)	
> = 4	348(59.30)	239(40.70)	1.00	
**Knowledge of important of using latrine.**				
Yes	609(52.80)	544(47.20)	2.93(1.80,4.80)	
No	26(27.70)	68(72.30)	1.00	
**Knowledge of cause of diarrheal diseases**				
Yes	610(54.30)	514(45.70)	4.65(2.89,7.53)	
No	25(20.30)	98(79.70)	1.00	
**Using leftover food**				
Yes	286(35.20)	527(64.80)	0.13(0.10,0.18)	
No	349(80.40)	85(19.60)	1.00	
**Knowledge of liquid waste to cause diseases**				
Yes	559(55.80)	442(44.20)	2.83(2.08,3.85)	1.95(1.23,3.08) ^a^
No	76(30.90)	170(69.10)	1.00	1.00
**Availability of latrine**				
Yes	515(75.00)	172(25.00)	10.90(8.35,14.45)	2.14(1.33,3.47) ^a^
No	120(21.40)	440(78.60)	1.00	1.00
**Availability of functional hand washing facility**				
Yes	185(91.10)	18(8.90)	13.57(8.06, 23.12	3.61(1.86,7.02) ^a^
No	450(43.10)	594(56.90)	1.00	1.00
**Availability of liquid wastes disposal pit**				
Yes	272(85.80)	45(14.20)	9.44(6.62,13.49)	2.23(1.39,3.63)^a^
No	363(39.00)	567(61.00)	1.00	1.00
**Availability of solid wastes disposal pit**				
Yes	396(74.20)	138(25.80)	5.69(4.41,7.35)	
No	239(33.50)	474(66.50)	1.00	
**Condition of the compounds**				
Clean	503(45.50)	603(54.50)	0.06(0.02,3.22)	
Not clean	132(93.60)	9(6.40)	1.00	

^a^ p ≤0.01

^b^ p<0.05

## Discussion

The study revealed that 51% households were practiced good food hygiene practices. Good food hygiene practices had variation between model (79.00%) and non model households (37.00%). This might be due to the health extension workers gave more attention to implementation of food hygiene and safety measure package to model households than non model households.

Among the study participants only 5% households (13% model and 0.20% non model households) prepared their food in the separated kitchen. This finding is smaller than the study conducted in Damboya district [13].This variation might be due to the fact that socio-economic and cultural variation of the study areas.

The finding of this study showed that model households were practiced good food hygiene practices than non model households. This could be model households were more trained about food hygiene and safety measures than non model households by the Health Extension workers.

Female household heads had practiced good food hygiene practices than male household heads. This might be due to the fact that in the study area culture, the responsibilities of food hygiene handling practices were given to females. Moreover, the health extension workers frequently met the female household heads.

Households who had latrine facilities had good food hygiene practices than households who had not latrine. This might be due to fact that households relate the importance of latrine with other packages including food hygiene practices.

The households who had liquid wastes disposal pit had two times good food hygiene practices than households who had not liquid wastes disposal pit. This could be due to the fact that household behavioral change of healthy risk of indiscriminate unsafe disposal of liquid waste.

The households who know liquid waste can cause disease had practiced two times good food hygiene practices than the households who don’t know liquid waste can cause a disease. This might be the fact that households’ knowledge of liquid waste disposal has directly related with good food hygiene practices.

The households who had functional hand washing facility practiced good food handling practice four times than the house hold had not have hand washing facility. This could be the understanding of the linkage of food hygiene handling practice with water supply and safety packages.

## Conclusion

From households in Abobo district only half of the household had practiced good food hygiene practices. The type of households, sex, knowledge of liquid waste to cause diarrheal diseases, availability of latrine, availability of functional hand washing facility and availability of liquid waste disposal site were identified as the factors associated with food hygiene practices. Health extension workers should play a great role in educating households regarding food hygiene practices to improve their knowledge and practices of the food hygiene.

## Supporting information

S1 File Questionnaire document. (DOCX)
