# Supplementary material for: Food hygiene practices and its associated factors among model and non model households in Abobo district, southwestern Ethiopia: Comparative cross-sectional study
Source: PLoS One. 2018 Apr 5;13(4):e0194391. doi: 10.1371/journal.pone.0194391 (PMC5886398; doi:10.1371/journal.pone.0194391)
Supplement: S1 File — (DOCX) [file pone.0194391.s001.docx]

## Questionnaire

## English Version

Questionnaires for assessment of Food Hygiene Practices and its Associated Factors among Model and non Model Households in Abobo District, Southwestern Ethiopia: Comparative Cross-sectional Study

Identification

Questionnaire identification number-------------------------------------

Name of HH head----------------------------Type of HH: 1.Model HH 2. None model HH

Kebele-------------------------

Date----------------------- Time started-------------------- Time ended---------------------

Questionnaire address Socio-demographic characteristics, knowledge of WSH and behaviors, environmental factors, and food handling hygiene

| S.no | Question | Possible Response | Code | Skip to---- |
| --- | --- | --- | --- | --- |
| 001 | Sex | 1. Female 2. Male |  |  |
| 002 | Age | ---------------Years |  |  |
| 003 | Education Level | Illiterate  Only read and write  Primary school(1-8)  Secondary school(9-12)  Diploma and Above |  |  |
| 004 | Religion | Orthodox  Protestant  Catholic  Muslim  Other(specify) |  |  |
| 005 | Marital status | Single  Married  Divorced  Widowed  Separated |  |  |
| 006 | Occupation | Farmer  Merchant  Civil servant  Daily Laborer  Other(Specify) |  |  |
| 007 | Ethnicity | Anywa  Kembata  Amhara  Other(Specify) |  |  |
| 008 | Family size in number | ---------------------------- |  |  |
| 009 | HHs income per month in Birr | --------------------------- |  |  |

| 010 | Do you wash your hand before eating regularly? | Yes  No |  |  |
| --- | --- | --- | --- | --- |
| 011 | When do you wash your hands? (don’t mention the options to the respondents) | Before preparing or cooking food  After visiting toilet  Before eating food  Before feeding children  After cleaning a house  After cleaning child’s bottom  Other (specify) |  |  |
| 012 | Do you know the type of diseases that can be prevented using hand washing? | Yes  No |  |  |
| 013 | If Q 012 is yes, can you mention some of them?  (don’t mention the options to the respondents) | Diarrhea  Typhoid  Amoeba  Giardia  Others (specify) |  |  |
| 014 | Do you know the importance of using latrine?  If yes, specify  ………………………..  ………………………… | Yes  No |  |  |
| 015 | Do you know children’s faece can transmit disease?  If yes, mention  ………………………..  ……………………… | Yes  No |  |  |
| 016 | Do you know the causes of diarrheal disease? | Yes  No |  | 017 |
| 017 | If Q 16 is yes, the causes of diarrheal disease (don’t mention the options to the respondents) | Contaminated food  Contaminated water  Dirty hands  Flies  Others(specify) |  |  |
| 018 | Do you know the preventive methods of diarrheal diseases? | Yes  No |  | 019 |
| 019 | If Q 18 is yes, the preventive method of diarrheal diseases (don’t mention the options to the respondents) | Keeping water safe  Keeping food clean  Using latrine  Washing hands  Other(specify) |  |  |
| 020 | Do cooked foods are covered? (ask the Head of the household to observe whether the material is covered or not) | Yes  No |  |  |
| 021 | How often do you cover cooked foods? | Always  Some times  Never |  |  |
| 022 | Have you ever used leftover food? | Yes  No |  | 023 |
| 023 | If Q 22 is yes, do you know leftover food can cause diseases? | Yes  No |  |  |
| 024 | Do you know solid waste can cause diseases?  If yes, mention  ………………………..  ………………………… | Yes  No |  |  |
| 025 | Do you know liquid waste can cause diseases? | Yes  No |  |  |

| 026 | Does household have water in home for 24 hrs?( Observational) | Yes  No |  |  |
| --- | --- | --- | --- | --- |
| 027 | Does household have latrine? | Yes  No |  | 028 |
| 028 | If Q 027 is yes, is the latrine used? (Observational) | Yes  No |  |  |
| 029 | Do you have functional hand washing facility? | Yes  No |  |  |
| 030 | Does household have liquid wastes disposal pit? | Yes  No |  |  |
| 031 | Does household have solid wastes disposal pit? | Yes  No |  |  |
| 032 | Hygienic condition of the compound of the house hold | Scattered refuse is seen  Scattered defecation seen  Free from refuse  Free from defecation  Other (specify) |  |  |

| 033 | Where the food is prepared? | With in the living quarter  In the kitchen  Outdoor  Other (specify) |  | 048,049, 050 |
| --- | --- | --- | --- | --- |
| 034 | If the answer of Q 047 is in the kitchen, does the kitchen have chimney? | Yes  No |  |  |
| 035 | If the answer of Q 047 is in the kitchen, how is the cleanness of the kitchen? | Clean  Not clean |  |  |
| 036 | If the answer of Q 047 is in the kitchen, does the kitchen quarter shared with animals? | Yes  No |  |  |
| 037 | Where the catering utensils are kept after cooking? | Kept on the shelf  Kept on the ground  Other(specify) |  |  |
| 038 | What do you use to clean utensils? | Cold water only  Hot water only  Either hot/cold water with soap  Water with Ash  Other (specify) |  |  |
| 039 | What type of water is used to clean utensils? | Clean water (the same as for drinking purpose?  Rain water  Other (specify) |  |  |
| 040 | Are cooked and raw foods kept separately (support with observation) | Yes  No |  |  |
| 041 | Are cooked foods covered? (observational) | Yes  No |  |  |
| 042 | Do you wash your hands before preparing food? | Yes  No |  |  |
| 043 | How often do you wash hands before cooking | Always  Some times |  |  |

Thank you for giving your time

Name of data collector-----------------------------date--------------Signature------------

Name of Supervisor -------------------------------date---------------Signature------------

Questionnaire-Anywa version

KØLEJ MAR ØT- JAATH KI MO CAANYE MAR JÖÖT DËËL

Gïr pïëc kiper rang mak pïï ki mak tøng mar cam mar jø paac moa nägo tïïö ni möödelli ki moa näk kär tïïö ni möödelli, Ya atuda Abwöbö, Løø kanyo päth cängngi yie ki Ithoopiea.

Ngïï

Kwään ngïï mar gïr løk pïëc man -------------------------------------

Nyen ngata bëënni yipaac ---------------------------

Teeng jøw paac: 1.Jø paac mo nägo tïïö ni möödel □ 2. Jø paac moa näk kär tïïö ni möödel □

Kabale-------------------------

Nïne-------------------Caa mana täge yie------------- Caa mana thume yie---------------

Jap dwätö ki ööny maal ki jammi moa näk guta dhaanhø mo jiy mo pëënynyï, yi jöör mak pïï ni beer ki jap yi bëët dhaanhø kiper yi jöör mak pïï mo jey moo bëënni yi paac, Pïëc kiper jappa atut, Pïëc kiper mak cam ni tøng mar jø paac.

| Kw. | Pïëc | Mana näk tïmö ni løk pïëc | Ngïï/code | Päär ri--- |
| --- | --- | --- | --- | --- |
| 001 | Dhak/Cwøw | Dhaagø 2. Dicwøø |  |  |
| 002 | Cwiiri mooi | Cwiiri mo --------------- |  |  |
| 003 | Lïng øt-göör | Kär göödö  Ngäya kwäänö ki göör keere  Ogat göör mana dikwøng(1-8)  Riet Ogat göör (9-12)  Dïplööma ki maal |  |  |
| 004 | Øt-Jwøk man lam | Ørthødøk  Pørøtïcthaan  Kaathilek  Mucelem  Mør(caani) |  |  |
| 005 | Lïng nywöm | Kär nyöömmö/nywømø  Onyöömmö  Geno opääö ki nywöm  Cwøre/Cïëë othøw  Ge bëëdö geno pääö ki bëëte |  |  |
| 006 | Tïïc mari | Ngat puur  Ngat nyigadha  Ngat tïïc mara akwöma  Tïïë mo bat  Mør(caani) |  |  |
| 007 | Wï-jur mari | Anywaa  Kambaatha  Amäära  (Mør(caani) |  |  |
| 008 | Kwään jø paac | ---------------------------- |  |  |
| 009 | Kwään Bïïrri mo joot jø-paaci ki yi dwääy | --------------------------- |  |  |

| 010 | Cengngi lwøgi lwøø cooth? | Kare  Pakare |  |  |
| --- | --- | --- | --- | --- |
| 011 | Cengngi lwøgi gø i wäne?(Kär løk pïëc mooi caani ji jey moo løk pïëc man) | Kanyo poode ni cam kär jiingngø wala kär thaalø  Kanyo dwöö ki øt-laac  Dikwøng ni cam poot kär camø  Dikwøng noo obwöre poot kär caamø  Kanyo lønyø ki jwiec øtø  Kanyo lønyø ki pooc thar nyilaal  Mør(caani) |  |  |
| 012 | Teeng täwe moa näk dëël da mänø ki gi ki ri lwøk ceng, ngäyi? | Kare  Pakare |  | 013 |
| 013 | Näk løk pïëc mar ri 012 bee kare ,løny man cäänni ki täwe mwøa ka mo nøk jaak?( Kär løk pïëc mooi caani ji jey moo løk pïëc man) | Täw døøreea  Täw thaypøøt  Täw amiippa  Täw jaardiia  Møøk(caani) |  |  |
| 014 | Ki man køny dëël ki øt-laac køny mare ngäyi?  Näk løk pïëc bee kare, caani  ………………………..  ………………………… | Kare  Pakare |  |  |
| 015 | Laac moa døøngngø mo bwöre, ngäyi ni løny man mwööe ki twäwe?  Näk løk pïëc bee kare, cäänni ki teeng täwe moa ka  ………………………..  ……………………… | Kare  Pakare |  |  |
| 016 | Jöö man ö täwe mo leth ec ki ge ngäyi? | Kare  Pakare |  | 017 |
| 017 | Näk løk pïëc mar ri 016 bee kare, jöö mano ö täwe mo leth ec ki regi angøøni? ( Kär løk pïëc mooi caani ji jey moo løk pïëc man) | Cam mo oränynyö  Pïï mo oränynyö  Ceng mo cøl  Lwangngi  Mør (caani) |  |  |
| 018 | Jïëdhi moo män dëël ki täwe mo leth ec ki ge ngäyi? | Kare  Pakare |  | 019 |
| 019 | Näk løk pïëc mar ri 018 bee kare, jïëdhi moo män dëël ki täwe mo leth ec ki ge ( Kär løk pïëc mooi caani ji jey moo løk pïëc man) | Gwøk pïï ni wøp  Gwøk cam ni tøng  Køny dëël ki øt-laac  Lwøk ceng  Mør (caani) |  |  |
| 020 | Caammi moa näk oløny ki thaal orïëbö?  (Pëëny ngata bëënni paac ki man rangngo jammi wala orïbö wala kär rïëbö) | Kare  Pakare |  |  |
| 021 | Ateeng kwöre adïï orïëppi caammi moa nägo othaalø? | Cooth cooth  Ki kwör møøk jaak  Ba rïëp ni bäre |  |  |
| 022 | Da caami mo dhöthø mo dëël ola køny ki geni en? | Kare  Pakare |  | 023 |
| 023 | Näk løk pïëc mar ri 022 bee kare, ngäyi ni caammi moo dhöthi kännö ki täwe? | Kare  Pakare |  |  |
| 024 | Jammi moa reyø moa nägo otal ngäyi ni kännö ki täwe? Näk løk pïëc bee kare, caan nyeng täwe mwøa ka  ………………………..  ………………………… | Kare  Pakare |  |  |
| 025 | Jammi moa reyø moa näk thec ngäyi ni kännö ki täwe? Näk løk pïëc bee kare, caan nyeng täwe mwøa ka.  ………………………..  ………………………… | Kare  Pakare |  |  |

| 026 | Jø paac pïï dagø jïgi yi paac kiper caae mo 24?(Nut mare wur rangi) | Kare  Pakare |  |  |
| --- | --- | --- | --- | --- |
| 027 | Jø paac øt-laac dagø jïgï? | Kare  Pakare |  | 028 |
| 028 | Näk løk pïëc mar ri 027 bee kare, øt-laac manø gø dëël di kønyø gø ennø?(ki man näk tïïö rangi) | Kare  Pakare |  |  |
| 029 | Kar lwøk ceng mo tïïö dagø? | Kare  Pakare |  |  |
| 030 | Buur mar kar wet jaami moa reyø moa näk thec dagø jï jø paac? | Kare  Pakare |  |  |
| 031 | Buur mar kar wet jaami moa reyø moa nägo otal dagø jï jø paac? | Kare  Pakare |  |  |
| 032 | Tøng mar yi kal mar jø paac | Juu mo keethø dagø ma ajoot yie  Laac moa døøngngø dagø jaak  Bung juu ni bäre  Laac moa døøngngø bung yie ni bare  Mør (caani) |  |  |

| 033 | Cam jiingngi kaa ngø? | Yi thääng kar bëëtö  Yi øt-thaal  Wøk jaak  Mør(caani) |  | 048,49,050 |
| --- | --- | --- | --- | --- |
| 034 | Näk løk pïëc mar ri 047 bee øt-thaal gïno døny jïre wøk ki yie dagø ri øt-thaal? | Kare  Pakare |  |  |
| 035 | Näk mo løk pïëc mar ri 047 bee yi øt-thaal, tøng mar øt-thaal nyïëdi? | Tøng  Ba tong |  |  |
| 036 | Näk mo løk pïëc mar ri 047 bee yi øt-thaal,øt-thaal thäängnge dinywangø ki lääy mo paac naa aciel? | Kare  Pakare |  |  |
| 037 | Jap thaal cïbï kaa kanyo løny thaalli? | Cïba kanyo cïp jammi yie  Cïba piny  Mør(caani) |  |  |
| 038 | Jammi mo thaal tïï ni tøng ki ngøøni? | Pïï moa ngec keere  Pïï moa lëëthö keere  Wala pïï moa lëëthö wala Pïï moa ngec ki caabuun  Pïï ki bur  Mør (caani) |  |  |
| 039 | Ateeng pïï mooe o tïïc jammi mo øt-thaal ni tøng ki gø? | Pïï mo tøng(dïët ki moo maath)  Pï køth  Mør (caani) |  |  |
| 040 | Caammi moa nägo thaalø ki moa näk poot kär thaalø ocïp piny noo opääö? | Kare  Pakare |  |  |
| 041 | Caammi moa näk oløny ki thaal,orïëbö?(wur rangi) | Kare  Pakare |  |  |
| 042 | Cengngi lwøgi lwøø kanyo poode ni cam poot kari jiingngø? | Kare  Pakare |  |  |
| 043 | Ateeng kwöre adïï olwøgi cengngi? | Cooth cooth  Ki kwör møøk jaak |  |  |

Yïïna pwøøa kiper mana cïppï caa mari

Nyeng ngato coong luup mooi--------------------------nïne------------------ngïï------------

Nyeng ngato rang tïïc man-------------------------------nïne---------------ngïï------------
